# *Ceiba pentandra* ethyl acetate extract improves doxorubicin antitumor outcomes against chemically induced liver cancer in rat model: a study supported by UHPLC-Q-TOF-MS/MS identification of the bioactive phytomolecules

**DOI:** 10.3389/fphar.2024.1337910

**Published:** 2024-02-02

**Authors:** Mohamed A. A. Orabi, Mohamed E. Abouelela, Faten M. M. Darwish, Mohamed S. A. Abdelkader, Bakheet E. M. Elsadek, Ahmed Abdullah Al Awadh, Mohammed Merae Alshahrani, Abdulaziz Hassan Alhasaniah, Nayef Aldabaan, Reda A. Abdelhamid

**Affiliations:** ^1^ Department of Pharmacognosy, College of Pharmacy, Najran University, Najran, Saudi Arabia; ^2^ Department of Pharmacognosy, Faculty of Pharmacy (Boys), Al-Azhar University, Cairo, Egypt; ^3^ Department of Pharmacognosy, Faculty of Pharmacy, Assiut University, Assiut, Egypt; ^4^ Department of Pharmacognosy, Faculty of Pharmacy, Sohag University, Sohag, Egypt; ^5^ Department of Biochemistry and Molecular Biology, Faculty of Pharmacy, Al-Azhar University, Assiut, Egypt; ^6^ Department of Clinical Laboratory Sciences, College of Applied Medical Sciences, Najran University, Najran, Saudi Arabia; ^7^ Department of Pharmacology, College of Pharmacy, Najran University, Najran, Saudi Arabia; ^8^ Department of Pharmacognosy, Faculty of Pharmacy, Al-Azhar University, Assiut, Egypt

**Keywords:** *Ceiba pentandra*, antitumor, *in vivo*, AFP-l3, hepatocellular carcinoma, doxorubicin, UHPLC-Q-ToF-MS/MS, dietary flavonoids

## Abstract

Hepatocellular carcinoma (HCC) is a prevalent cancer worldwide. Late-stage detection, ineffective treatments, and tumor recurrence contribute to the low survival rate of the HCC. Conventional chemotherapeutic drugs, like doxorubicin (DOX), are associated with severe side effects, limited effectiveness, and tumor resistance. To improve therapeutic outcomes and minimize these drawbacks, combination therapy with natural drugs is being researched. Herein, we assessed the antitumor efficacy of *Ceiba pentandra* ethyl acetate extract alone and in combination with DOX against diethylnitrosamine (DENA)-induced HCC in rats. Our *in vivo* study significantly revealed improvement in the liver-function biochemical markers (ALT, AST, GGT, and ALP), the tumor marker (AFP-L3), and the histopathological features of the treated groups. A UHPLC-Q-TOF-MS/MS analysis of the *Ceiba pentandra* ethyl acetate extract enabled the identification of fifty phytomolecules. Among these are the dietary flavonoids known to have anticancer, anti-inflammatory, and antioxidant qualities: protocatechuic acid, procyanidin B2, epicatechin, rutin, quercitrin, quercetin, kaempferol, naringenin, and apigenin. Our findings highlight *C. pentandra* as an affordable source of phytochemicals with possible chemosensitizing effects, which could be an intriguing candidate for the development of liver cancer therapy, particularly in combination with chemotherapeutic drugs.

## 1 Introduction

Hepatocellular carcinoma (HCC) is a multistage, widely occurring disease; in Egypt, it is the sixth most common in women and the second most common in men (Rashed et al., 2020). The prevalence of risk factors is linked to environmental, dietary lifestyle factors as aflatoxins, alcohol (Omar et al., 2013) and pathologic factors like diabetes and viral hepatitis (McGlynn and London, 2005). Numerous studies indicate that these risk factors may work in concert or separately to affect the chance of developing liver cancer (McGlynn and London, 2005; Rashed et al., 2020).

Herbal medicines are being used more and more as anticancer therapies since they are readily available and have fewer adverse effects than conventional drugs. Several natural compounds, including resveratrol, curcumin, quercetin, silibinin, silymarin, *n*-*trans*-feruloyl octopamine, lycopene, gallic acid, berberine emodin, and phloretin, have shown promise in treating HCC and other hepatic toxicities (Rawat et al., 2018; Mandlik and Mandlik, 2021).

The natural products studied for their impact on HCC appear to have a variety of mechanisms to attain their effects, including promoting apoptosis and cell cycle arrest, exhibiting antioxidant and anti-inflammatory properties, boosting the immunity, suppressing angiogenesis, preventing cancer cell invasion, and detoxifying liver carcinogens (Mandlik and Mandlik, 2021).

*Ceiba pentandra* (L.) Gaertn. (Bombacaceae) trees are planted for horticultural purposes in many countries. Its aerial parts, leaves, stems, and stem-barks showed anti-inflammatory, hepatoprotective, antioxidant, hypoglycemic, hypolipidemic, bactericidal, and anticancer properties in pharmacological studies (Lim, 2012; Refaat et al., 2014; Abouelela et al., 2019). In terms of phytochemistry, it is distinguished by the presence of anthocyanins, steroids, triterpenes, sesquiterpenes, and other polyphenols, some of which are featured in our earlier work (Abouelela et al., 2018; Abouelela et al., 2020).

Among *Ceiba* species, *C. pentandra* is the most studied for its cytotoxic and antitumor effects. The bark extracts increased the mean survival time of tumor-bearing mice in Ehrlich ascites carcinoma (EAC, liquid tumor) and Dalton’s ascites lymphoma (solid tumor) models (Kumar et al., 2016). It also showed a potent cytotoxic effect on mouse melanoma (B16-F10) and EAC cell lines (Kumar et al., 2016). In a study on angiogenesis, the leaf extract drastically reduced the number of tubes that human umbilical vein endothelial cells could generate (Nam et al., 2003). The formation of reactive oxygen species (ROS) associated the oxidative stress has been shown to enhance tumor growth. This supports the importance of antioxidants to safeguard cells from oxidative stress and prevent DNA damage, while their anticancer properties can inhibit the growth and proliferation of cancer cells (Kola et al., 2022). This was demonstrated in our previous study, where *C. pentandra* ethyl acetate (EtOAc) extract significantly reduced methotrexate-induced kidney damage in rats, which was attributed to the involvement of the association of antioxidant and anti-inflammatory, and antiapoptotic mechanisms (Abouelela et al., 2020).

One of the most widely used approaches in the treatment of unresectable HCC in recent decades has been the use of standard chemotherapeutic drugs like doxorubicin (DOX) (Lahoti et al., 2012). Nevertheless, DOX’s low efficacy—likely a consequence of drug resistance—as well as its undisputed involvement in the onset of numerous dose-related side effects that ultimately lead to inadequate antitumor outcomes—have limited its clinical applicability (Patel et al., 2010). As a result, increasing both the therapeutic effectiveness and the DOX index is regarded as an unmet medical need.

Significant evidence suggests that co-therapy with drugs from natural source is an accepted attempt in cancer therapy, as it can attain improved therapeutic effects than an individual drug or modality while also reducing side effects and drug resistance (Capone et al., 2014). Therefore, research into the use of chemosensitizing phytochemicals in conjunction with approved chemotherapeutic drugs is being conducted as a substitute and superior method of managing and treating cancer (Wagner, 2011).

The current study aimed to explore the likely use of *C. pentandra* EtOAc faction as a therapeutic agent and/or a co-therapy with DOX for HCC based on an *in vivo* assessment of the extract’s efficacy in inhibiting tumor growth in chemically induced HCC in a rat model. Consequently, we have analyzed the EtOAc extract by an ultra-high-performance liquid chromatography-electrospray ionization-quadrupole-time of flight-mass spectrometer (UHPLC/Q-TOF/MS/MS) to explore the compounds accountable for the antitumor activity. Molecular ions and their fragment ions (MS/MS data) were detected during the LC/MS profiling of secondary metabolites. These were then located in databases and compared with existing literature to ensure accurate identification (Dunn et al., 2013).

## 2 Materials and methods

### 2.1 The extraction and fractionation

Aerial parts (2 kg), involving leaves and tender young branches, of *C. pentandra* were gathered from the garden of the Faculty of Agriculture, University of Assiut, Egypt. The air-dried plant material was extracted by soaking in MeOH/H_2_O (8:2, *v/v*, 5 L × 3) at normal temperature. The aqueous methanolic extract was dried at ≤ 45°C under vacuum until reached a constant weight (250 g). The obtained extract was partitioned between water/methylene chloride (MC), EtOAc, and *n*-butanol, successively, and afford the corresponding MC (52 g), EtOAc (28 g), *n*-butanol (19 g), and water fractions (14a g), respectively (Abouelela et al., 2018).

### 2.2 Evaluation of *C. pentandra* EtOAc extract’s antitumor effects against artificially generated HCC in rat model

#### 2.2.1 Animals and experimental design

The experiment was conducted using healthy male Wistar rats (14–15 weeks old, weighing 140–150 g) obtained from Assiut University, Egypt. The rats were housed in controlled conditions with standard diet and water access. The rats were randomly divided into a control group (n = 8) receiving carboxymethyl cellulose (CMC) and an experimental group (n = 32) administered diethylnitrosamine (100 mg/L) in their water *ad libitum* for 8 weeks, the latter was further subdivided one-month post-diethylnitrosamine (DENA). The subgroups included a DENA control group (n = 8), a DENA+DOX group (n = 8) receiving weekly intravenous doxorubicin (4 × 2.5 mg/kg, I.V.), a DENA+Extract group (n = 8) with daily oral plant extract (400 mg/kg) gavages, and a DENA+DOX+Extract group (n = 8) receiving daily oral treatments of both DOX+Extract concurrently for 4 weeks (Atwa et al., 2021).

A week afterward the last treatment, all animals were submitted to cervical decapitation under isoflurane anaesthesia. For preparing the serum, blood samples were taken from all rats via the retro-orbital vein plexus. After autopsy, the rat livers were excised, cleansed of adhering connective tissues and fat, then cleaned in ice-cold isotonic saline, and finally classified into three divisions. The first was stored in 10% neutral buffered formalin solution and examined histopathologically, while the other two portions were immediately flash-frozen in liquid N_2_ and kept separately at −70°C for following biochemical assays (Di Stefano et al., 2008; Mansour et al., 2019).

#### 2.2.2 Biochemical estimations

Non-enzymatic liver functions, such as albumin and total bilirubin, were estimated using colorimetric assay kits according to the manufacturer’s instructions. Serum ALT, AST, GGT, ALP levels were estimated using the procedures and kits outlined by the manufacturers (Elitech diagnostic Co., France). Moreover, serum AFP-L3, a specific tumour marker for HCC, was estimated using a rat-specific ELISA assay kit following the manufacturer’s protocol (Elabscience Biotechnology Co., Ltd. Wuhan, China) (Fathy et al., 2017).

#### 2.2.3 Histopathological examination

For histopathological inspection, liver tissues were embedded in paraffin, fixed in 10% formalin, and consistently stained with haematoxylin and eosin (H&E) stain. After cleansing in a sterile tap water, the tissues were dehydrated using serial dilutions of methanol, ethanol, and absolute ethanol. In a hot air oven, samples were cleaned in xylene and embedded in paraffin for 24 h at 56°C. Paraffin beeswax tissue blocks were used for making tissue slices at four microns using a sled microtome. A glass slide was used to hold tissue samples, which were then deparaffinized and stained with H&E stain for examination under a light microscope (Culling, 2013).

#### 2.2.4 Statistical analysis

Utilizing the version 5 of the GraphPad Prism (Graph Pad Software, San Diego, United States), data statistical analysis was accomplished. Analysis of variance (ANOVA) and Tukey’s *t*-test were considered to compare the results. *p* < 0.05 was the accepted level of significance, and the data were graphically represented as mean SD.

### 2.3 Identification of the *C. pentandra* EtOAc’ compounds by the UHPLC-Q-TOF-MS/MS analysis

#### 2.3.1 Chemicals and materials

Methanol (CH_3_OH) and acetonitrile (CH_3_CN) of HPLC quality (Merck, Germany), formic acid (Fisher, United States). Culture plastic dishes and plates (96-well) were bought from Becton Dickinson (Franklin Lakes, NJ, United States).

#### 2.3.2 UHPLC-Q-TOF-MS/MS instruments

UHPLC ExionLC™ AC system, involving dual high-pressure gradient pump, vacuum degasser, autosampler, column oven (SCIEX, United States), coupled with AB SCEIX Triple-TOF ™ 5600^+^ mass spectrometer (AB SCIEX, United States, with ESI source), and KQ-300DB-type CNC ultrasonic instrument (Kunshan Ultrasonic Instrument Co., Ltd.). BSA224S-CW-type electronic balance (Sedolis Scientific Instrument Company). LG16-W-type high-speed centrifuge (Beijing Jingli Centrifuge Co., Ltd.).

#### 2.3.3 Sample preparation for UHPLC-Q-TOF-MS/MS analysis

A sample of the EtOAc fraction (2 mg) was dissolved in 5 mL of dissolution system [deionized H_2_O/CH_3_OH/CH_3_CN (5:2.5:2.5, *v*/*v*)] to obtain 0.4 μg/μL working solution. The solution was vortexed for 2 min followed by ultrasonication for 10 min and centrifuge for 5 min at 1792 g. An aliquot (10 μL) was injected for an LC analysis system along with a 10 μL dissolution system as a control.

#### 2.3.4 Chromatographic conditions for UHPLC-Q-TOF-MS/MS analysis

The UHPLC-Q-TOF-MS/MS analysis was conducted according to chromatographic conditions by Abdelhafez, et al. (2018). Briefly, 10 μL of the sample was injected into the UHPLC ExionLC™ AC system, with pre-column in-line filter disk (0.5 µm × 3.0 mm) (Phenomenex, United States), Xbridge C_18_ column (2.1 mm × 50 mm, 3.5 µm) (Waters, United States), at 40°C. The mobile phase is composed of deionized H_2_O (A) and CH_3_CN (B) each containing 0.1% formic acid. The gradient elution started with 10% B, which linearly increased to 90% B within 20 min and remained isocratic for 5 min before linearly decreasing back to 10% B for the following 3 min. The mobile phase was then equilibrated for 10 min between injections. The flow rate was set at 0.3 mL/min.

#### 2.3.5 Mass spectrometry conditions for UHPLC-Q-TOF-MS/MS analysis

The ESI-Q-TOF-MS in positive ion mode was used in the following conditions: The ESI ion source voltage was 5–500 V, the ion source temperature was 550°C, the cracking voltage was 80 V, and the collision energy was 35 eV, respectively. The collision energy spread was ±15 eV, the atomizing gas was nitrogen, the nebulizer gas was 45 kPa, the heater gas was 45 kPa, and the curtain gas was 25 kPa. The primary mass spectrometer has a scan range of mass-to-charge ratio (*m/z*) 50 to 1,000 for ESIMS. Information dependent acquisition sets the peaks with a response value exceeding 200 cps for secondary mass spectrometry. The sub-ion scan range was *m*/*z* of 50–1,000, and dynamic background subtraction was turned on and ions tolerance was 10 ppm with the exclusion of former target ions with 3 repeats and 3 s and exclusion of isotopes within 2.0 *Da* (Abdelhafez et al., 2018).

#### 2.3.6 Data analysis for UHPLC-Q-TOF-MS/MS results

The data acquisition software used for processing raw data is Analyst TF 1.7.1 software (AB SCEIX, United States). Data processing software systems: Peakview Ver. 2.2, and Markerview Ver. 1.3 (AB SCEIX, United States) was used for peaks extraction from total ion chromatogram with a signal-to-noise ratio >3 (non-targeted analysis). They were also used for peak filtering based on comparing the peak intensity of plant sample to blank (intensity ratio >3), and for removing isotopic peaks. Accurate mass and composition for the precursor and fragment ions were analyzed using Peakview software integrated with the instrument. The first and second-order spectra were compared with accurate mass and fragmentation patterns of metabolites in online databases: mass bank; a dictionary of natural products; metlin databases; mzCloud; human metabolome database (HMDB); PubChem; competitive fragmentation modeling for components identification (CFM-ID version 2.0), combined with the literature on previously isolated compounds from the family, to determine the structures of the detected compounds.

## 3 Results

### 3.1 Antitumor activity of *C. pentandra* EtOAc extract

Despite the advances made recently in HCC treatment, there has been limited success in improving the survival rates of people with HCC (Ganesan and Kulik, 2023). This is partly due to the lack of effective treatment options, the recurrence of tumors and the tendency to identify the disease at an advanced stage (Han et al., 2023). Therefore, the object of the current study was to evaluate the *in vivo* anticancer efficacy of *C. pentandra* extract, either alone or in combination with the conventional DOX, against diethylnitrosamine (DENA)-induced HCC in rats.

Our results revealed that animals in DENA group exhibited obvious impaired liver functions including substantial hypoalbuminemia (*p* < 0.01), together with elevated serum activities of alanine transaminase (ALT) (*p* < 0.001), aspartate aminotransferase (AST) (*p* < 0.001), gamma-glutamyl transferase (GGT) (*p* < 0.001) and alkaline phosphatase (ALP) (*p* < 0.001), and total serum bilirubin level (*p* < 0.001), in comparison to the healthy control group—see Table 1. DENA-induced changes in serum indicators of liver function may be due to peroxidation of hepatocyte membranes, resulting in increased release of AST, GGT, ALT, ALP, and total bilirubin from compromised liver tissues as has been previously demonstrated in various models of DENA-induced hepatocellular deterioration (Bansal et al., 2005; El-Hawary et al., 2015).

**TABLE 1:** Effect of tested *C. pentandra* extract on liver function tests and tumor markers of rat models.

	Control	DENA	DENA+DOX	DENA+Extract	DENA+Extract+DOX
Albumin (g/dL)	3.81 ± 0.37	2.88 ± 0.48^**^	2.77 ± 0.63^**^	2.98 ± 0.25^*^	3.77 ± 0.67^†,§§,‡^
sALT (IU/L)	18.2 ± 8.61	101.6 ± 33.7^***^	84.3 ± 36.4^***^	79.0 ± 22.9^***^	42.3 ± 10.0^†††,§,‡^
sAST (IU/L)	78.0 ± 22.7	315.4 ± 78.4^***^	280.5 ± 78.8^***^	238.0 ± 72.0^***^	135.4 ± 77.4^†††,§§,‡^
sGGT (IU/L)	19.5 ± 8.51	98.7 ± 39.9^***^	116.3 ± 28.9^***^	99.2 ± 30.1^***^	50.5 ± 25.1^†,§§§,‡^
ALP (IU/L)	71.1 ± 22.7	298.5 ± 99.8^***^	233.9 ± 75.7^***^	202.4 ± 65.3^**,†^	104.0 ± 34.4^†††,§§,‡^
T. Bilirubin (mg/dL)	0.35 ± 0.17	1.43 ± 0.49^***^	1.15 ± 0.61^**^	1.11 ± 0.22^**^	0.52 ± 0.31^†††,§,‡^
AFP-L3 (ng/mL)	13.9 ± 4.40	284.4 ± 54.8^***^	260.0 ± 83.0^***^	216.4 ± 81.0^***^	134.0 ± 58.2^***,†††,§§§,‡^

Data are presented as mean ± SD (n = 8). *, †, §, and ‡ indicate significant changes from control, DENA, DENA+Extract, and DENA+DOX, groups respectively. *, †, §, and ‡ indicate significant change at *p* < 0.05; **, ††, §§, and ‡‡ indicate significant change at *p* < 0.01; ***, †††, §§§, and ‡‡‡ indicate significant change at *p* < 0.001.

The present research noted that although the administration of either *C. pentandra* extract or DOX is moderately effective in restoration of these impaired indicators to their respective normal ranges, the administration of *C. pentandra* alone has a comparatively stronger effect than the standard chemotherapeutic doxorubicin against chemically induced HCC in a rat model. Meanwhile, a positive outcome emerged in the group that received a combined treatment of *C. pentandra* extract and DOX (DENA+Extract+DOX), leading to a noticeable enhancement in the overall estimated liver function indices as illustrated in Table 1. The animals treated with the combined regimen (DENA+Extract+DOX) displayed a significant rise in serum albumin levels (*p* < 0.05). Additionally, they exhibited noteworthy reductions in serum ALT (*p* < 0.001), AST (*p* < 0.001), GGT (*p* < 0.05), ALP (*p* < 0.001), and total bilirubin levels (*p* < 0.001). Remarkably, the combination therapy outperformed individual treatments in the (DENA+Extract) and (DENA+DOX) groups. This superiority was evident through a significant surge in serum albumin levels (*p* < 0.05 and *p* < 0.01, respectively), coupled with considerable reduction in serum ALT (*p* < 0.05 and *p* < 0.05, respectively), AST (*p* < 0.05 and *p* < 0.01, respectively), GGT (*p* < 0.05 and *p* < 0.001, respectively), ALP (*p* < 0.05 and *p* < 0.01, respectively), and total bilirubin levels (*p* < 0.05 and *p* < 0.05, respectively).

The results of the assessments of the isoform L-3 of alpha-fetoprotein (AFP-L3), an HCC tumour marker, provided additional evidence that supports the superiority of *C. pentandra* extract and DOX combination over both *C. pentandra* extract and DOX individual administration. Several investigators have recently used this marker for the timely recognition and observing of treatment response, as well the HCC relapse, and/or malignance invasion, as well as surrogate markers of the liver clinicopathological changeability with suitable sensitivity and specificity (Lagana et al., 2013; Jain, 2014; Wang et al., 2014).

According to our findings, the serum AFP-L3 level in the DENA group was significantly higher than in the normal healthy control group (*p* < 0.001) (Table 1). Although each of *C. pentandra* extract and DOX alone failed to significantly reduce the DENA-induced elevation in AFP-L3 levels, their co-administration efficiently reduced the elevated AFP-L3 levels, approximately 52.8% (*p* < 0.001), 38.0% (*p* < 0.05), and 48.4% (*p* < 0.001) decreases in comparison to the DENA, DENA+Extract, and DENA+DOX groups, respectively.

**FIGURE 1:**
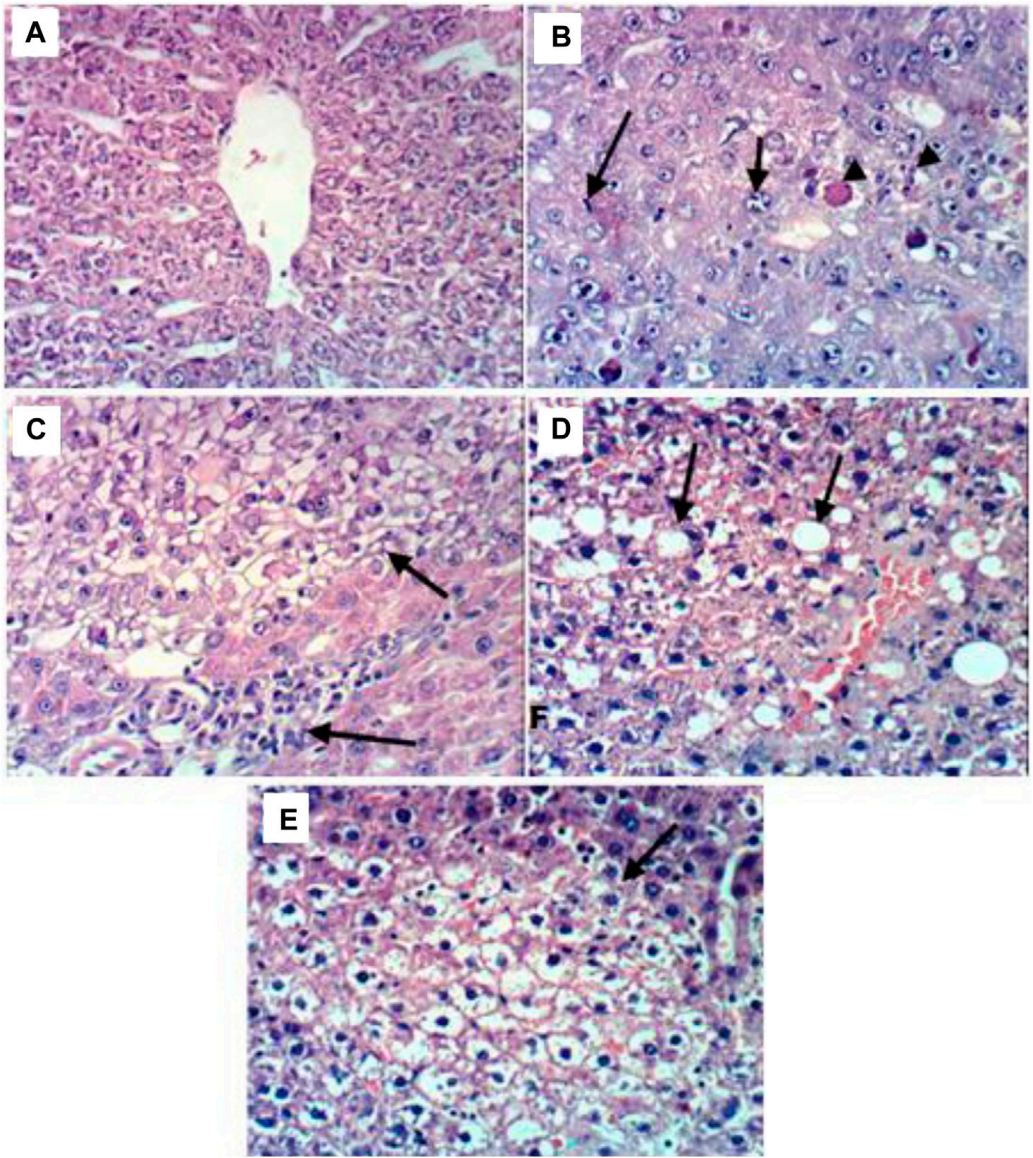
Representative photomicrographs of liver sections from different groups. **(A)** Liver tissues of the control group showing the normal histological structure of hepatic lobule; **(B)** Liver tissues of the DENA group revealing HCC in which carcinoma cells showed large vesicular nuclei with more than one nucleoli, mitotic figure, and intracytoplasmic eosinophilic hyaline bodies; **(C)** Liver tissues of the DENA+extract group showing relative improvement in the histopathological picture as the examined sections revealed only small focal clear hepatocytes; **(D)** Liver tissues of the DENA+DOX group showing severe histopathological alterations confined as HCC with destruction in the normal trabecular structure of the liver and proliferation of oval cells with mitotic figures; **(E)** Liver tissues of the DENA+extract+DOX group showing marked improvement in the histopathological picture with steatosis and vacuolation of hepatocytes, dilatation and congestion of hepatic sinusoids.

The liver tissues histological investigation (Figures 1A–E) offered strong proof for the combined C. pentandra extract and DOX’s valuable effects in fighting DENA-induced HCC. The representative photomicrographs of liver sections obtained from animals in the DENA group revealed HCC in which carcinoma cells showed large vesicular nuclei with more than one nucleoli, mitotic figure, and intracytoplasmic eosinophilic hyaline bodies (Figure 1B). Analogous histological findings have been previously documented in published DENA-induced rat models (Afzal et al., 2012; Golla et al., 2013).

Meanwhile, the rat’s liver administered the combination of *C. pentandra* extract and DOX, on the other hand, showed relative enhancement in the histopathological features. The investigated sectors showed steatosis and hepatocytes vacuolation, dilatation, and hepatic sinusoidal congestion (Figure 1E). These findings definitely imply the tumor-defeating ability of the mix involving both *C. pentandra* extract and DOX against DENA-induced HCC. Meantime, livers of rats treated with DOX alone showed severe histopathological changes confined as HCC with normal trabecular destruction of the liver structure and propagation of oval cells with mitotic figures (Figure 1D). However, the livers of the rats administered *C. pentandra* EtOAc extract alone showed relative improvement in the histopathological features as the examined sectors showed only small focal clear hepatocytes (Figure 1C).

**FIGURE 2:**
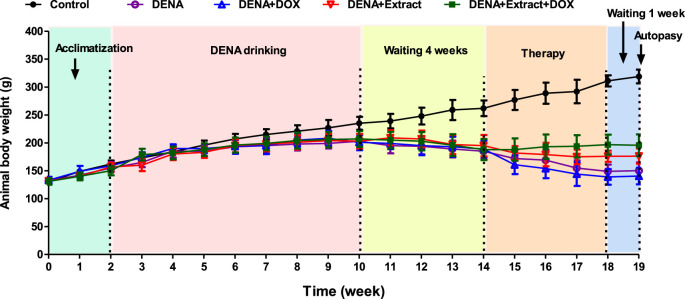
Illustration of the changes in the animal’s body weights in different groups during the experiment.

Worthwhile, throughout the experiment, healthy control group rats stayed vital, and conscious, and achieved a significant increase of the body weight as compared to the DENA-drinking animals, which appeared sluggish with an insensible increase in the body weight. As shown in Figure 2, all DENA-drinking rats suffered weight loss during the exposure before treatment, which is probably due to the initial tumor burden or the tumor metastasis. Similar findings were previously seen in the same animal model (Bansal et al., 2005; Elsadek et al., 2017). Groups treated with extracts or therapy displayed stable weight trends. The DENA+DOX and DENA+Extract+DOX groups experienced initial sharp weight declines, followed by gradual recovery, while the DENA drinking group had a slight dip. This result represents respectable evidence of the superiority of the ethyl acetate extract of *C. pentandra* over the conventional DOX regarding the systemic toxicity and tolerability. Overall, this data visually illustrates the impact of different treatments on animal body weight, aiding researchers in concluding their effects.

In consistence with our findings, *C. pentandra* bark extract has shown a protective effect on CCl_4_-induced liver damage and oxidative stress (Ellappan et al., 2022), and a similar protective effect against paracetamol-induced hepatotoxicity in rats in previous studies (Bairwa et al., 2010). However, our investigation today is more comprehensive.

To explore the phytochemical constituents corresponding to this promising antitumor activity, a UHPLC-Q-TOF-MS/MS analysis of the *C. pentandra* EtOAc extract was conducted.

### 3.2. Analysis of the EtOAc extract using UHPLC-Q-TOF-MS/MS

**FIGURE 3:**
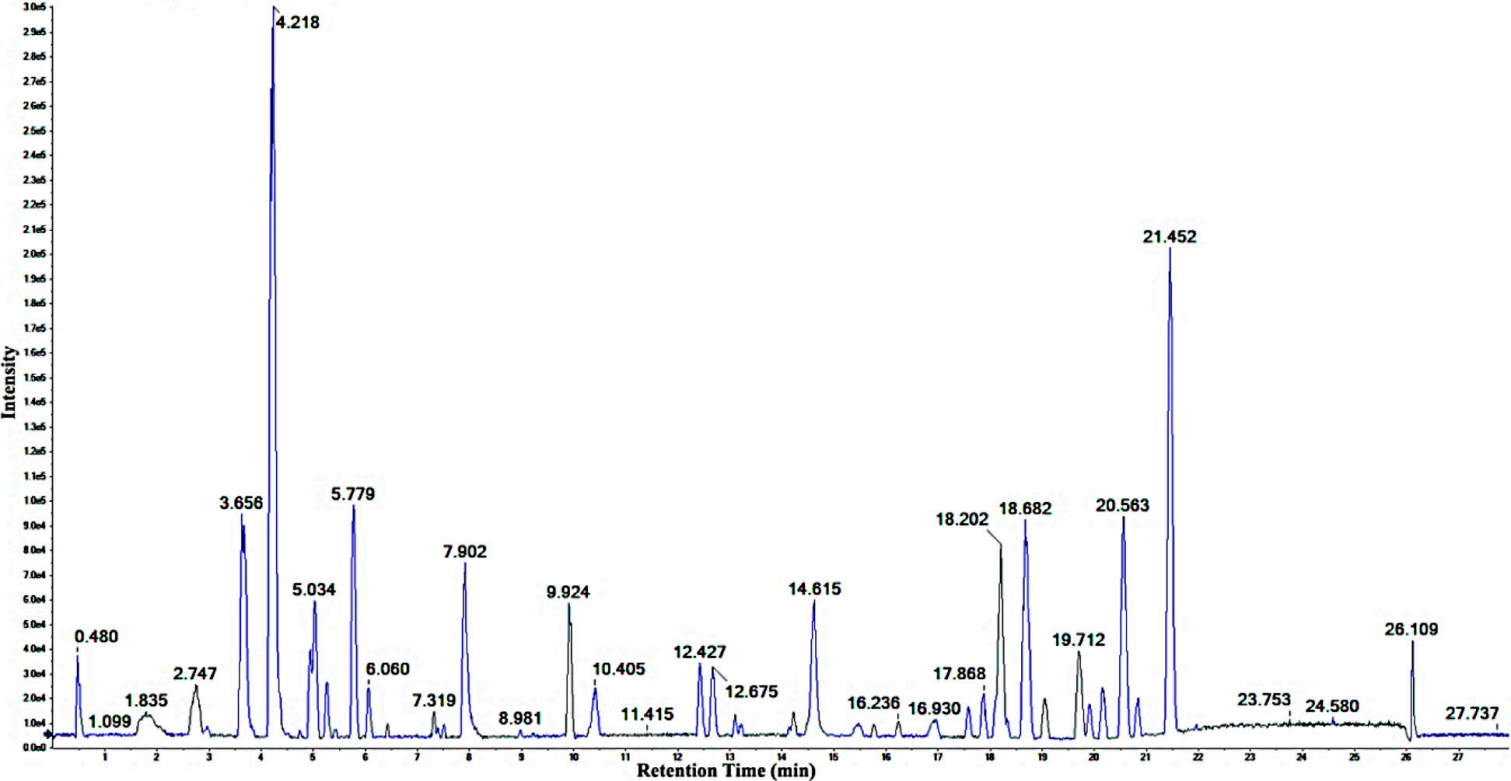
The positive ion TIC of the ethyl acetate extract of *C. pentandra*.

Following optimizing the chromatographic and mass spectroscopic conditions, a UHPLC-Q-TOF-MS/MS (positive ion mode) investigation was conducted. The total ion current (TIC) map of the compounds of the EtOAc extract was produced (Figure 3). The marker view 1.3 software’s non-targeted screening revealed that several different chemicals are represented by each chromatographic peak. Based on Q-TOF-HRESIMS measured ions mass (at < 1.0 × 10^−5^ deviation) combined with the isotope abundance ratio, the molecular formula for each common peak of the compounds was accurately determined. The peak was accurately located by molecular ion (*m/z*) value search regardless of the retention time drifts due to chromatographic parameters or instrument equipment changes, which ensures good reproducibility of the metabolite’s fingerprint.

Structural analysis of the components was attained by two-stage mass spectrometry to obtain the accurate element composition and characteristic fragment ions for each compound. The resulting data were then matched with the calculated and reported high-resolution mass of known compounds, and fragmentation patterns for the given classes of compounds, in connection with the structural information given by the online databases and the secondary mass spectrum (MS^2^) analysis of the previously isolated compounds available in literature (Refaat et al., 2013).

**FIGURE 4:**
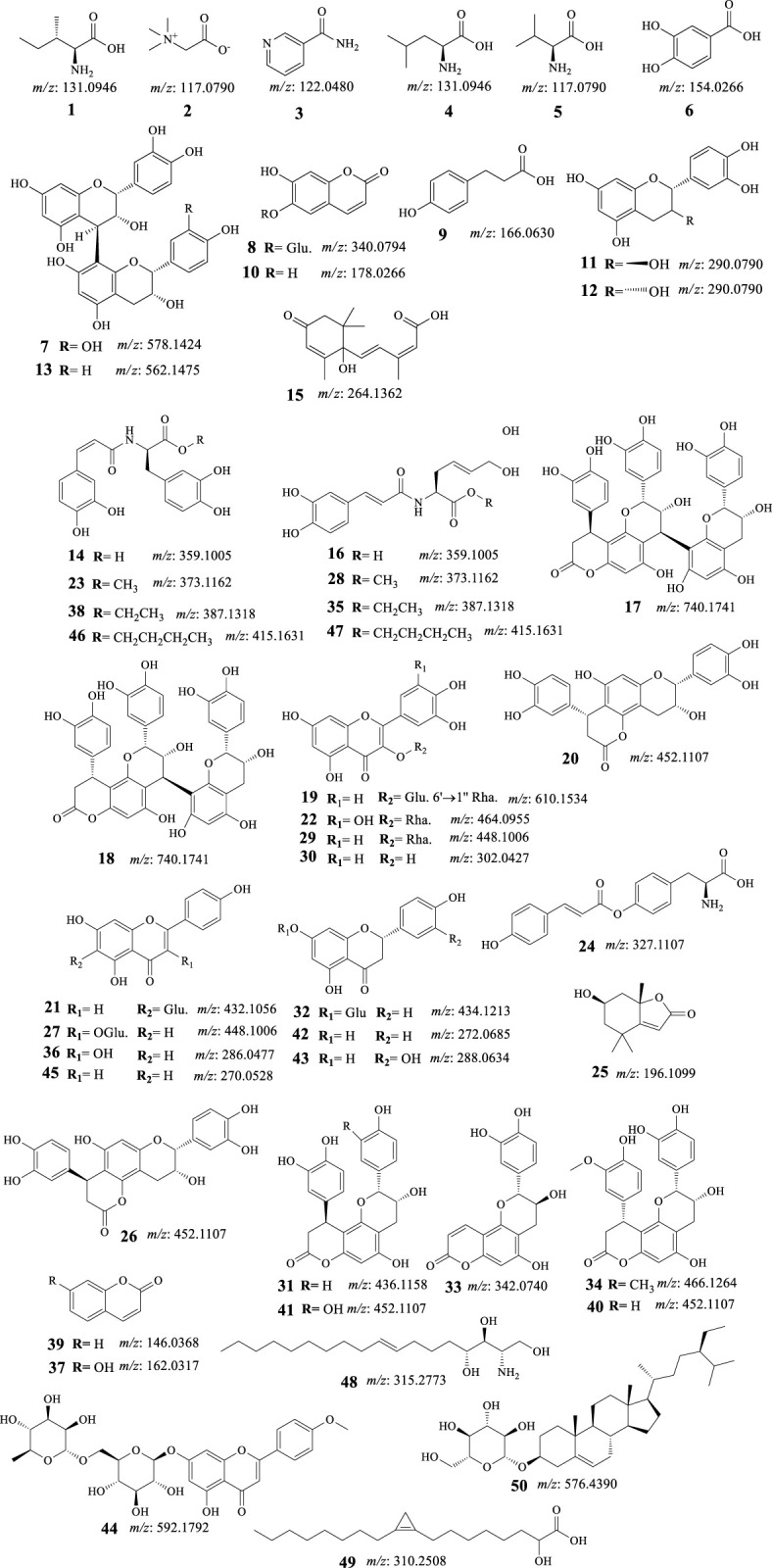
Structures of identified phytomolecules of *C. pentandra* ethyl acetate fraction.

Fifty components (Table 2; Figure 4) were identified by analysis of the mass results. The retention time, the high-resolution mass of the molecular ion, chemical formula, and MS^2^ fragments of the identified chemical constituent are shown in Table 2. The compounds were arranged according to their retention time. The identified phytoconstituents are four amino acids (**1**, **2**, **4**, and **5**), nine amino acids derivatives (**14**, **16**, **23**, **24**, **28**, **35**, **38**, **46**, and **47**), fourteen flavonoids (**11**, **12**, **19**, **21**, **22**, **27**, **29**, **30**, **32**, **36**, **42**, **43**, **44**, and **45**), eight flavanolignans (**17**, **18**, **20**, **26**, **31**, **34**, **40**, and **41**), two procyanidins (**7** and **13**), five coumarins (**8**, **10**, **33**, **37**, and **39**), two phenolic acids (**6** and **9**), and six miscellaneous compounds (**3**, **15**, **25**, and **48**–**50**). The coumarin aesculetin and the flavonoids apigenin, astragalin, and rutin are reported in the plant for the first time.

**TABLE 2:** List of identified phytomolecules in *C. pentandra* ethyl acetate extract.

No.	*R*_t_ (min)	Formula	M wt	MS^1^ [M+H]^+^	Error (ppm)	MS^2^	Identified compound	References
			*m/z*	*m/z*		*m/z*		
1	0.47	C_6_H_13_NO_2_	131.0946	132.1012	9.89	—	Isoleucine	He et al. (2018)
2	0.49	C_5_H_11_NO_2_	117.0790	118.0857	9.57	—	Betaine	He et al. (2018)
3	0.54	C_6_H_6_N_2_O	122.0480	123.0550	7.56	106, 80, 78, 53, 51	Niacinamide	Smith et al. (2005)
4	0.54	C_6_H_13_NO_2_	131.0946	132.1026	−0.47	—	Leucine	He et al. (2018)
5	0.56	C_5_H_11_NO_2_	117.0790	118.0858	9.09	—	Valine	He et al. (2018)
6	0.88	C_7_H_6_O_4_	154.0266	155.0333	7.55	120, 113, 93, 72, 67, 65	Protocatechuic acid	Lin et al. (2015)
7	1.13	C_30_H_26_O_12_	578.1424	579.1494	1.59	453, 427, 409, 291, 289, 287, 273, 271, 259, 247, 163, 139, 127	Epicatechin-(4*β*→8)-epicatechin	Maldini et al. (2009)
8	1.15	C_15_H_16_O_9_	340.0794	341.0875	−0.47	265, 242, 201, 179, 161, 137, 123	Aesculin	Tine et al. (2017)
9	1.16	C_9_H_10_O_3_	166.0630	167.0700	5.30	149, 125, 123, 121, 91, 84, 77	Dihydro-*p*-coumaric acid	Narváez-Cuenca et al. (2012)
10	1.44	C_9_H_6_O_4_	178.0266	179.0339	3.24	161, 151, 135, 133, 123, 117, 105, 89, 77	Aesculetin	Tine et al. (2017)
11	1.57	C_15_H_14_O_6_	290.0790	291.0867	0.94	272, 249, 214, 207, 165, 161, 147, 139, 123	Catechin	Tsimogiannis et al. (2007)
12	1.63	C_15_H_14_O_6_	290.0790	291.0873	−1.20	273, 214, 207, 165, 161, 147, 139, 123	Epicatechin	Tsimogiannis et al. (2007)
13	1.91	C_30_H_26_O_11_	562.1475	563.1554	−0.02	517, 435, 427, 411, 409, 393, 291, 287, 275, 273, 231	Epicatechin-(4*β*→8)-epiafzelechin	Gu et al. (2003)
14	1.94	C_18_H_17_NO_7_	359.1005	360.1078	1.59	342, 224, 198, 181, 178, 163, 152, 145, 135, 117, 89	*cis*-Clovamide	Locatelli et al. (2013), Masike et al. (2017)
15	2.10	C_15_H_20_O_4_	264.1362	265.1440	0.34	247, 229, 200, 172, 160, 141, 135, 131, 123, 119, 105	Abscisic acid	Smith et al. (2005)
16	2.63	C_18_H_17_NO_7_	359.1005	360.1078	1.64	342, 224, 198, 181, 178, 163, 152, 145, 135, 117, 107, 89	*trans*-Clovamide	Locatelli et al. (2013), Masike et al. (2017)
17	3.17	C_39_H_32_O_15_	740.1741	741.1821	−0.11	589, 571, 451, 433, 409, 341, 289, 179, 163	Cinchonain IIa	Hokkanen et al. (2009)
18	3.21	C_39_H_32_O_15_	740.1741	741.1814	0.86	589, 571, 451, 433, 409, 341, 289, 179, 163	Cinchonain IIa isomer	Hokkanen et al. (2009)
19	3.40	C_27_H_30_O_16_	610.1534	611.1598	2.44	465, 303	Rutin	Moqbel et al. (2018)
20	3.46	C_24_H_20_O_9_	452.1107	453.1182	0.93	411, 343, 313, 301, 259, 191, 163, 147, 123	Cinchonain Ic	Hokkanen et al. (2009), Abouelela (2016)
21	3.53	C_21_H_20_O_10_	432.1057	433.1127	1.85	415, 397, 379, 367, 337, 313, 283	Isovitexin	El-Sayed et al. (2017)
22	3.55	C_21_H_20_O_12_	464.0955	465.1012	4.67	319, 303, 214, 147	Myricitrin	Moqbel et al. (2018)
23	3.59	C_19_H_19_NO_7_	373.1162	374.1241	−0.09	359, 342, 332, 314, 238, 212, 195, 178, 163, 153, 152, 135, 117	*cis-Clovamide* methyl ester	Peña-Morán et al. (2016)
24	3.78	C_18_H_17_NO_5_	327.1107	328.1171	4.45	147, 119, 91	*p*-Coumaroyl tyrosine	Clifford and Knight (2004)
25	3.82	C_11_H_16_O_3_	196.1099	197.1175	1.84	179, 161, 135, 133, 115, 107, 105, 91, 79	Loliolide	Smith et al. (2005)
26	3.96	C_24_H_20_O_9_	452.1107	453.1187	−0.24	435, 411, 343, 325, 317, 313, 301, 259, 191, 163, 137, 123	Cinchonain Id	Hokkanen et al. (2009), Abouelela (2016)
27	4.08	C_21_H_20_O_11_	448.1006	449.1083	0.28	287	Astragalin	March et al. (2006)
28	4.21	C_19_H_19_NO_7_	373.1162	374.1238	0.88	359, 332, 238, 212, 195, 178, 163, 153, 152, 145, 135, 117, 89	*trans-Clovamide* methyl ester	Allen (2016), Peña-Morán et al. (2016)
29	4.32	C_21_H_20_O_11_	448.1006	449.1076	1.93	303, 287, 229, 153, 147, 129, 85, 71	Quercitrin	Moqbel et al. (2018)
30	4.38	C_15_H_10_O_7_	302.0427	303.0504	0.54	285, 274, 257, 229, 201, 165, 153, 149, 137, 121	Quercetin	Cuyckens and Claeys (2004)
31	4.42	C_24_H_20_O_8_	436.1158	437.1239	−0.53	419, 395, 343, 327, 301, 191	Corbulain Ia	Wungsintaweekul et al. (2011)
32	4.55	C_21_H_22_O_10_	434.1213	435.1282	2.26	358, 273, 179, 153, 147	Prunin	Moqbel et al. (2018)
33	4.65	C_18_H_14_O_7_	342.0740	343.0806	3.56	311, 221, 191, 147, 123	Epiphyllocoumarin	Gyobin (2016)
34	4.72	C_25_H_22_O_9_	466.1264	467.1344	−0.18	449, 421, 343, 327,301, 203, 191, 147, 89	Smiglabrone B	Xu et al. (2013), Gu et al. (2015)
35	4.74	C_20_H_21_NO_7_	387.1318	388.1393	1.10	371, 358, 209, 191, 177, 163, 149, 145, 117	*cis-Clovamide* ethyl ester	Won et al. (2004), Kim et al. (2015)
36	5.01	C_15_H_10_O_6_	286.0477	287.0556	0.02	258, 231, 213, 153, 121	Kaempferol	Cuyckens and Claeys (2004)
37	5.07	C_9_H_6_O_3_	162.0317	163.0392	2.26	145, 135, 117, 107, 89, 77, 63	Umbelliferone	Tine et al. (2017)
38	5.27	C_20_H_21_NO_7_	387.1318	388.1399	−0.55	371, 358, 343, 209, 177, 163, 149, 145, 134, 117, 89	*trans-Clovamide* ethyl ester	Won et al. (2004), Kim et al. (2015)
39	5.30	C_9_H_6_O_2_	146.0368	147.0442	3.20	119, 118, 103, 91, 65	Coumarin	Tine et al. (2017)
40	5.31	C_24_H_20_O_9_	452.1107	453.1185	0.38	435, 411, 343, 325, 317, 313, 301, 259, 191, 163, 147, 137, 123	Cinchonain Ib	Hokkanen et al. (2009), Abouelela (2016)
41	5.45	C_24_H_20_O_9_	452.1107	453.1181	1.12	435, 411, 343, 317, 313, 301, 259, 191, 163, 137, 123	Cinchonain Ia	Hokkanen et al. (2009), Abouelela (2016)
42	5.56	C_15_H_12_O_5_	272.0685	273.0750	4.98	153, 147, 123	Naringenin	Fang et al. (2006)
43	5.64	C_15_H_12_O_6_	288.0634	289.0702	3.66	271, 259, 247, 229, 179, 163, 153, 145, 135, 117	Eriodictyol	Tsimogiannis et al. (2007)
44	6.13	C_28_H_32_O_14_	592.1792	593.1870	0.24	447, 285, 270, 242, 129	Linarin	Feng et al. (2014)
45	6.74	C_15_H_10_O_5_	270.0528	271.0609	−0.59	243, 229, 225, 163, 153, 145, 121, 119	Apigenin	Zhang et al. (2005)
46	7.37	C_22_H_25_NO_7_	415.1631	416.1703	1.70	398, 371, 360, 342, 254, 237, 181, 163, 152, 145, 135, 117	*cis-Clovamide* butyl ester	Allen (2016)
47	7.42	C_22_H_25_NO_7_	415.1631	416.1706	0.94	398, 371, 342, 254, 237, 181, 163, 152, 145, 139, 135, 117	*trans-Clovamide* butyl ester	Allen (2016)
48	11.6	C_18_H_37_NO_3_	315.2773	316.2847	1.74	298, 280, 262, 214, 135, 79, 60	Dehydrophytosphingosine	Smith et al. (2005)
49	17.4	C_19_H_34_O_3_	310.2508	311.2583	1.12	293, 251, 237, 177, 135, 123, 107, 97, 81, 67, 55	2-Hydroxysterculic acid	Smith et al. (2005)
50	21.6	C_35_H_60_O_6_	576.4390	577.4482	−2.30	—	*β*-Sitosterol-3-*O*-*β*-D-glucopyranoside	Smith et al. (2005)

## 4 Discussion

The occurrence and progression of liver cancer are well-known to be multi-gene, and multi-stage processes (Li et al., 2023a). Herbal extracts or natural substances extracted from them have been used to treat liver cancer patients in recent years. For example, curcumin’s several pharmacologic properties against HCC make it a useful treatment for the disease. In preclinical models of liver disease, silymarin, a flavonolignan complex found in milk thistle seeds, has been demonstrated to have hepatoprotective properties.

In our preceding phytochemical isolation from *C. pentandra* has evidenced the occurrence of large amount of flavonolignans in addition to flavonoids, phenylpropanoids (Abouelela et al., 2020). Pharmacological research of extracts from various morphological parts of *C. pentandra* have also demonstrated anti-inflammatory, hepatoprotective, antioxidant, hypoglycaemic, hypolipidemic, and anticancer (Lim, 2012; Refaat et al., 2014; Abouelela et al., 2019). In our *in vivo* study of *C. pentandra*, the ethyl acetate fraction has emerged as the most promising nephroprotective part of the extract.

To explore further health benefits about *C. pentandra*, *in vivo* anticancer efficacy of *C. pentandra* ethyl acetate extract, alone or in combination with the conventional DOX, against diethylnitrosamine (DENA)-induced HCC in rats was investigated. The results showed that the DENA-induced changes in serum indicators of liver function were relatively restored to their respective normal ranges by the administration of either *C. pentandra* extract or DOX. Although, the administration of *C. pentandra* alone had a comparatively stronger effect than DOX against chemically induced HCC in a rat model. Also, the rats treated with the ethyl acetate extracts displayed stable weight trends during the study, whereas the rats treated with DOX experienced sharp weight declines, which is a common side effect associated with DOX (Colombo et al., 1989). The combination of *C. pentandra* extract and DOX outperformed the individual treatments in the DENA+extract and DENA+DOX groups, leading to a noticeable enhancement in the overall estimated liver function indices. The DENA+DOX and DENA+Extract+DOX groups experienced initial sharp weight drops, followed by gradual recovery, while the DENA drinking group had a slight decline.

Furthermore, co-administration of C. pentandra extract and DOX effectively decreased the raised AFP-L3 levels, despite the fact that neither substance by itself was able to appreciably lower the DENA-induced rise in levels of the AFP-L3 HCC tumour marker. The liver tissues histological investigation offered strong proof for the combined *C. pentandra* extract and DOX’s valuable effects in fighting DENA-induced HCC.

In order to investigate the compounds responsible for the antitumor properties, we have consequently analyzed the EtOAc fraction using an UHPLC/Q-TOF/MS/MS. The mass analysis results (Table 1) highlighted the occurrence of large number of plant polyphenols, including fourteen flavonoids (**11**, **12**, **19**, **21**, **22**, **27**, **29**, **30**, **32**, **36**, **42**, **43**, **44**, and **45**), eight flavanolignans (**17**, **18**, **20**, **26**, **31**, **34**, **40**, and **41**), two procyanidins (**7** and **13**), five coumarins (**8**, **10**, **33**, **37**, and **39**), two phenolic acids (**6** and **9**).

Plant polyphenols demonstrate protective effects against oxidative stress, inflammation, ageing, and other pathophysiological processes in relation to liver disorders (Cha and DeMatteo, 2005; Wu et al., 2012; Sangineto et al., 2020; Ruiz-Manriquez et al., 2022). The protective effects of various polyphenols on liver cancer are caused by numerous molecular mechanisms involving modulation of lipid metabolism, glucose metabolism, mitochondrial metabolism, oxidative stress, and other metabolic reactions (Li et al., 2023a).

Hepatocellular carcinoma-preventive properties of flavonoids including those identified in the EtOAc fraction of *C. pentandra* via several intracellular signaling pathways have been explained in various reports (Xia et al., 2013; García et al., 2018). Among the reported antitumor molecular mechanisms of some of the identified compounds are as follows:

(i). Apigenin (**45**, Figure 4) has been shown to exhibit anticancer properties in various malignancies, including the HCC cell line (BEL-7402/ADM) through the miR-101/Nrf2 pathway (Gao et al., 2017)]. Hesperidin and apigenin strengthened the cytotoxic effect of DOX on the HepG2 cell line (Korga et al., 2019). It also retrieved the cytotoxicity of natural killer cells via repairing the linkage between cancer cells and NK cells and inhibiting the generation of regulatory T cells (Tregs) (Li et al., 2023b). Apigenin enhanced HIF-1α expressing HCC natural killer cytotoxicity through increasing the cluster of differentiation 95 (CD95)/CD95 ligand (CD95L) interaction (Lee and Cho, 2022).
(ii). Catechin (**11**, Figure 4) exhibited proliferation-inhibitory and pro-apoptosis effects on the human HepG2 cell line (Monga and Sharma, 2013). Catechin also induced cell apoptosis which was associated with inhibiting the expression of B-cell lymphoma 2 (Bcl-2) as well as elevating the expression of Bcl-2-associated X protein (BAX) and caspase-3 in a concentration-dependent manner (Liao et al., 2015).
(iii). Isovitexin [**21** apigenin-6-*C*-glucoside, Figure 4], suppressed the stemness of human HCC (SK-Hep-1) cells. It has revealed suppression of sphere and colony formation, decreased CD44^+^ cell populations, and decreased ATP binding cassette subfamily G member 2 (ABCG2), aldehyde dehydrogenase 1 family member A1 (ALDH1), and NANOG mRNA levels while increasing miR-34a levels (Xu et al., 2020).
(iv). Quercetin (**30**, Figure 4) has been shown to have anti-tumor effects on HCC through various mechanisms. One study found that quercetin can reverse multidrug resistance (MDR) of HCC cells by down-regulating the expression of mdr1, multidrug resistance-associated protein (MRP), glutathione-S-transferase-π (GST-π), and H-ras, while also down-regulating P-glycoprotein expression (Wei et al., 2012). Another study found that quercetin inhibits the migration ability of HCC cells by inhibiting the expression of transcriptional co-suppressor, C-terminal binding protein 1 (CtBP1), and up-regulating the expression of epithelial adhesion molecule E-cadherin, a tumor suppressor protein (Tang et al., 2018). Additionally, quercetin has been shown to inhibit the proliferation of glycolysis-addicted HCC cells by reducing hexokinase 2 and the Akt-mTOR pathway (Wu et al., 2019). Another study found that quercetin can inhibit the growth of transplanted HCC in nude mice by reducing Inositol trisphosphate (IP3) production and Bax protein expression (Huang and Zhang, 2001). Finally, quercetin-3-*O*-glucoside has been shown to induce human DNA topoisomerase II inhibition, cell cycle arrest, and apoptosis in HCC cells (Sudan and Rupasinghe, 2014)
(v). A study investigated the kaempferol (**36**, Figure 3) anti-migratory and anti-invasive effects on HCC cells (Ju et al., 2021) has been realized that kaempferol reduced these effects of HCC cells by targeting the matrix metalloproteinase-9 (MMP-9) protein kinase B (AKT) pathways. In addition, kaempferol lowered the MMP-9 protein expression and activities and suppressed the phosphorylation of the Akt expression. Another study showed that kaempferol can sensitize liver cancer cells toward the effect of sorafenib for the management of advanced HCC (Nair et al., 2020). Furthermore, kaempferol effectively inhibited cellular proliferation and migration, and enhanced cell cycle arrest, autophagy, apoptosis, and mediated chemosensitivity with 5-fluorouracil in HCC cells (Kannan et al., 2019)
(vi). Naringenin (**42**, Figure 4) has been found to reduce HCC cell viability, block epithelial-mesenchymal-transition, and inhibit sphere creation, cell migration and invasion. It also downregulated stemness-associated transcription factors and diminished hypoxia-inducible factor-1 (HIF-1) activity (Kang et al., 2019). Furthermore, naringenin substantially boosted the sensitivity of HCC cells to medicines, and hampered the growth of the HCC tumor, and metastasis of HCC cells to the lung. Naringenin silenced Wnt/β-catenin signaling by provoking β-catenin degradation and stopping its nuclear translocation. Upregulation of glycogen synthase kinase 3β (GSK3β) looked to be crucial for naringenin’s repressing effect in the signaling pathway (Kang et al., 2019)
(vii). A study performed by Wang et al. observed that eriodictyol (**43**, Figure 4) induced concentration-dependent selective cytotoxicity against the Hep-G2 cell line. Eriodictyol has also induced morphological changes, apoptosis-related chromatin condensation, and nuclear fragmentation. Furthermore, it also encouraged G2/M cell cycle arrest, downregulated Bcl-2 protein, and upregulated BAX and poly-ADP ribose polymerase (PARP) in these cells (Wang et al., 2016).
(viii). Protocatechuic acid (**6**, Figure 4) induced cell death in HepG2 cells through a c-Jun N-terminal kinase-dependent mechanism (Yip et al., 2006; Yip et al., 2006).
(ix). Kumar et al. developed a suitable delivery system for umbelliferone *β*-D-galactopyranoside (UFG) targeting the enhancement of its therapeutic efficacy against DENA-induced HCC in Wistar rats. The anticancer potential of these nanoparticles was able to manage DENA-induced reactive oxygen species generation, mitochondrial dysfunction, proinflammatory cytokines alteration, and induction of apoptosis (Kumar et al., 2017).
(x). Aesculetin (**10**, Figure 4) has shown promising anticancer activity against various organ cancers including breast, lung, and liver (Zhang et al., 2021). A study that investigated the anticancer properties of aesculetin against gastric cancer demonstrated that aesculetin lowered cellular proliferation in a time and dose-dependent manner, suppressed the clonogenic tendency of cancer cells, and encouraged apoptosis. The study also revealed that aesculetin blocked the phosphatidylinositol-3-kinase (PI3K)/protein kinase B (AKT)/mammalian target of rapamycin (mTOR) pathway in gastric cancer cells (Zhang et al., 2021).

Collectively, previous studies on the impact of identified components on the therapy of HCC provide credence to our experimental results. Hence, it is obvious that *C. pentandra* EtOAc extract is rich in promising phytoconstituents with known antioxidant, anti-inflammatory, and antitumor properties which may integrate to afford hepatoprotective activity and/or anti-HCC activity in combination with chemotherapeutics such as doxorubicin.

## 5 Conclusion

Our study demonstrates that the EtOAc fraction of *C. pentandra* hydromethanolic extract has a relatively respectable activity against chemically induced HCC in rat model. Moreover, it strongly suggests that the combinatorial use of *C. pentandra* extract and DOX produced undoubted *in vivo* antitumor activity against this type of cancer, as proven herein through estimation of diverse molecular, biochemical, and histological parameters. In addition, fifty phytomolecules have been identified from the same fraction based on UHPLCQ-TOF-MS/MS analysis. Numerous anti-cancer effects of flavonoids have been shown, including immune-modulating, anti-neoplastic, and drug-sensitizing capabilities. Among them, the dietary phenolic acid, protocatechuic acid **(6)**, flavonoids, catechin **(11)**, isovitexin **(21)**, quercetin **(30)**, kaempferol **(36)**, naringenin **(42)**, eriodictyol **(43)**, and apigenin **(45)**, and coumarin, aesculetin **(10)**, have been shown to fight cancer by various mechanisms (Liu-Smith and Meyskens, 2016; Lotha and Sivasubramanian, 2018; Hazafa et al., 2019; Ezzati et al., 2020), which agree with our discoveries.

Overall, despite the lack of a specific molecular mechanism study in this research, our findings highlight that *C. pentandra*, which is rich in flavonoids and the related promising compounds flavolignans, coumarins, and phenolic acids, could be a hopeful candidate for the development of liver cancer therapies in combination with doxorubicin. This will spur further preclinical and clinical research on this phytomolecules-rich plant as an inexpensive cancer care strategy when considering tailored, preventative, and predictive treatment.

## Data Availability

The original contributions presented in the study are included in the article/Supplementary Material, further inquiries can be directed to the corresponding authors.
