# Supplementary figures and images for: Ceiba pentandra ethyl acetate extract improves doxorubicin antitumor outcomes against chemically induced liver cancer in rat model: a study supported by UHPLC-Q-TOF-MS/MS identification of the bioactive phytomolecules
Source: Front Pharmacol. 2024 Feb 2;15:1337910. doi: 10.3389/fphar.2024.1337910 (PMC10871037; doi:10.3389/fphar.2024.1337910)

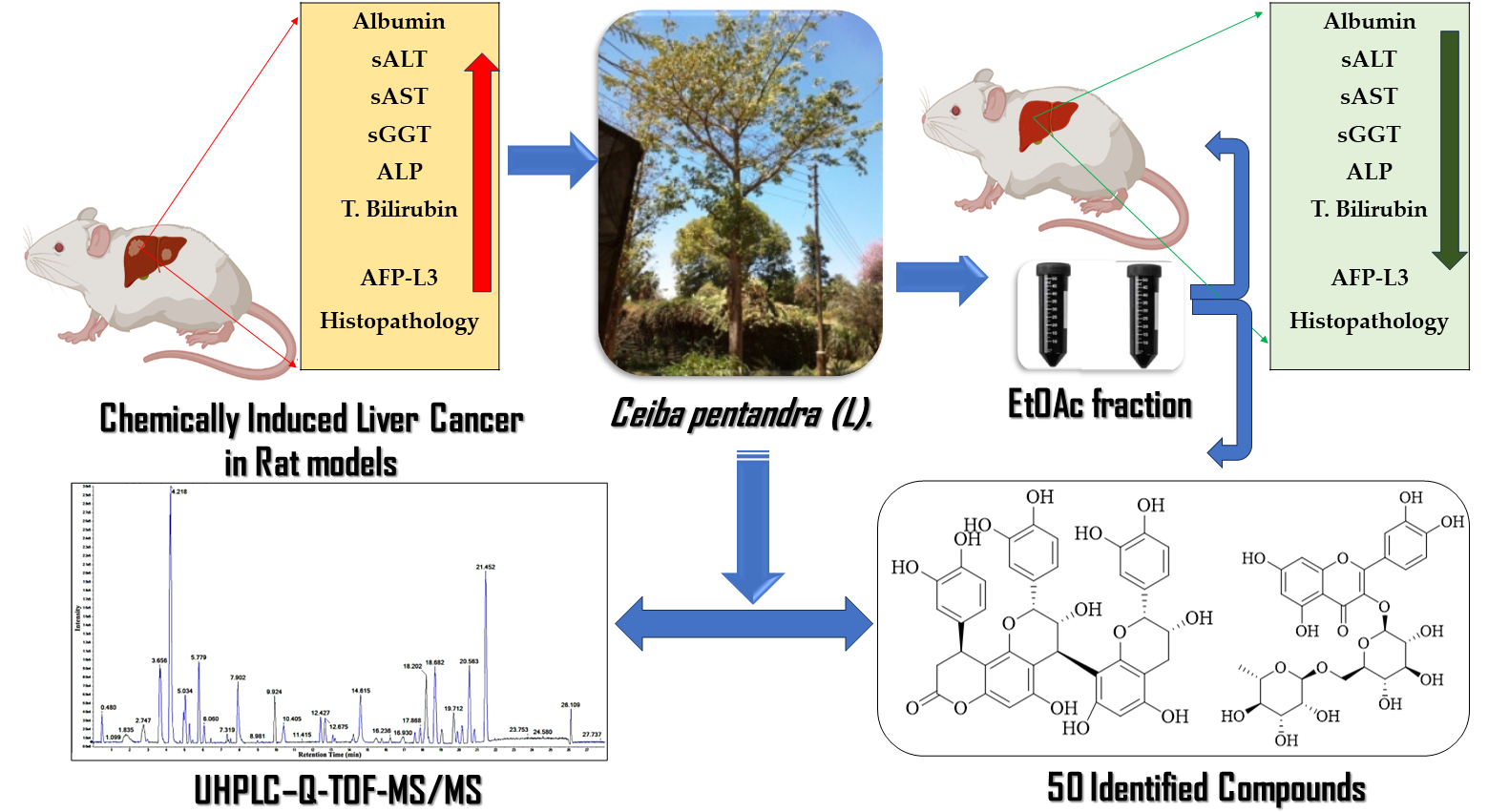

Supplement: Supplementary file 1 [file Image1.PNG]
